# Enhanced corrosion resistance and cytocompatibility of biodegradable Mg alloys by introduction of Mg(OH)_2_ particles into poly (L-lactic acid) coating

**DOI:** 10.1038/srep41796

**Published:** 2017-02-02

**Authors:** Yong-juan Shi, Jia Pei, Jian Zhang, Jia-lin Niu, Hua Zhang, Sheng-rong Guo, Zhong-hua Li, Guang-yin Yuan

**Affiliations:** 1 National Engineering Research Centre of Light Alloy Net Forming and State Key Laboratory of Metal Matrix Composite, Shanghai Jiao Tong University, Shanghai 200240, China; 2 School of Pharmacy, Shanghai Jiao Tong University, Shanghai 200240, China; 3 Microport Endovascular (Shanghai) Co., Ltd, Shanghai, 201318, China

## Abstract

A strategy of suppressing the fast degradation behaviour of Mg-based biomaterials by the introduction of one of Mg degradation products Mg(OH)_2_ was proposed according to the following degradation mechanism, Mg + 2H_2_O ⇋ Mg(OH)_2_ + H_2_↑. Specifically, Mg(OH)_2_ submicron particles were mixed into poly (L-lactic acid) (PLLA) to synthesize a composite coating onto hydrofluoric acid-pretreated Mg-Nd-Zn-Zr alloy. The *in vitro* degradation investigations showed that the addition of Mg(OH)_2_ particles not only slowed down the corrosion of Mg matrix, but also retarded the formation of gas pockets underneath the polymer coating. Correspondingly, cytocompatibility results exhibited significant improvement of proliferation of endothelial cells, and further insights was gained into the mechanisms how the introduction of Mg(OH)_2_ particles into PLLA coating affected the magnesium alloy degradation and cytocompatibility. The present study provided a promising surface modification strategy to tailor the degradation behaviour of Mg-based biomaterials.

Magnesium and magnesium alloys have been considered as a promising candidate for the next-generation biodegradable cardiovascular stent as it shows the possibility to degrade or be absorbed once fulfilling the aim of strutting a narrowed arterial vessel^1^,^2^. Their application is likely to overcome the limitations of permanent stents in growing young aged, particularly in newborns, considering the issues of growth^3^. Recently, the PROGRESS-AMS and DREAMS clinical trials sponsored by BIOTRONIK AG have shown promising results^4^,^5^,^6^. However, the too rapid corrosion/degradation of magnesium matrix, consequently resulting in the early loss of structural support way before the recovery of vascular function, has posed a huge challenge for the clinical use of magnesium alloy implants^2^,^6^,^7^. Thus, it is essential for magnesium and magnesium alloys to acquire adequate surface modifications/treatments to enhance corrosion resistance.

Among the varieties of surface modification techniques^7^,^8^,^9^,^10^,^11^,^12^,^13^,^14^,^15^,^16^, polymer coating presents as a facile and effective method for protecting magnesium alloy from rapid corrosion. An ideal polymer coating for biodegradable magnesium alloy implants is considered to own suitable mechanical properties, excellent biocompatibility and controlled degradation rate, apart from preventing magnesium substrate from too rapid corrosion, thus prolonging its life expectancy. Poly(L-lactic acid) (PLLA) is one of the several biodegradable polymers preferred for biomedical applications, of which the hydrolysis product lactic acid is found naturally in the body^10^. Several studies have reported that the corrosion rates of PLLA-coated Mg or Mg alloys decreased to certain extent compared with those of uncoated samples in DMEM or SBF, and exhibited enhanced cytocompatibility^10^,^17^,^18^. Nevertheless, the too rapid release of hydrogen from the corroding Mg substrate still often leads to the formation of gas pockets under the coating^19^, which may cause the coating spalling and peeling off the substrate, resulting in deleterious consequences for clinical use.

Essentially, the degradation process of magnesium alloy is a corrosion reaction of magnesium with water^20^:


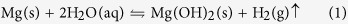


insoluble Mg(OH)_2_ is formed once the metal reaches direct contact with water, but it is not stable in the presence of anions like Cl^−^, SO_4_^2−^ or under acidic conditions^21^:


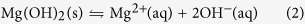


Considering the above mechanisms, several measures can be taken to impede the dissolution of the magnesium alloy: (1) to control the amount of the aggressive anions in the environment; (2) to increase the content of the reaction products, thus changing the reaction equilibrium and retarding the progress of the forward reaction; (3) providing a slightly alkaline environment to delay the forward reaction. Based on the above analyses (2) and (3), it can be hypothesized that the introduction of some Mg(OH)_2_ particles, whose chemical composition is identical to the degradation product of Mg matrix, into the PLLA coating as an additive, would be beneficial for enhancing the protection of coating against corrosion reaction.

In this paper, a Mg-Nd-Zn-Zr alloy (noted as JDBM) was used as the substrate material, whose biodegradable behaviours have been studied in previous works in our group^22^,^23^,^24^, showing a nanophasic uniform and slower corrosion behaviour compared to commercial magnesium alloys. Here, a composite PLLA coating mixed with Mg(OH)_2_ submicron particles was prepared on JDBM covered with a conversion layer generated by the pre-treatment with hydrofluoric acid (noted as HF) to provide short-term protection. To our knowledge, this is the first time reported to prepare a composite coating consisting of PLLA and Mg(OH)_2_ on magnesium alloy-based implant materials and to achieve a significant protective effect and good biocompatibility with this coating. The influence of the particles on the *in vitro* protective effect of the polymer coating as well as the corrosion mechanism was investigated and discussed. The effect on the cytocompatibility of the composite coating was quantitatively evaluated with endothelial cell line model.

## Results

### Physio-chemical characterizations of coatings

**Figure 1:**
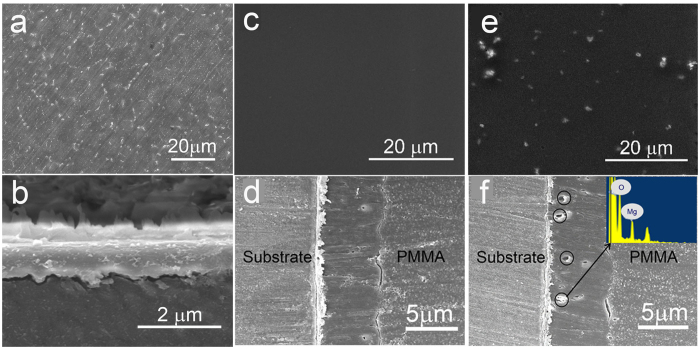
SEM images of HF, PLLA and HM/PLLA-coated samples. (**a**) top surface and (**b**) cross section of HF samples: (**c**) top surface and (**d**) cross section of PLLA-coated samples; (**e**) top surface and (**f**) cross section of HM/PLLA-coated samples.

Figure 1 depicts the SEM surface morphologies and cross-section view of HF samples^23^, the PLLA-coated samples (noted as PLLA) and Mg(OH)_2_/PLLA-coated samples (noted as HM/PLLA). On the HF sample (Fig. 1a and b), the MgF_2_ layer, with a thickness of ~1.5 μm, was generally flat except for some parallel lines, which are scratches resulting from the polishing process. The white particles distributing uniformly on the surface were second-phase particles mainly composed of Mg_12_Nd^25^. PLLA coating displayed uniform and smooth surface morphology as shown in Fig. 1c, while Mg(OH)_2_ particles distributed discretely in the HM/PLLA coating (white particles in Fig. 1e. The thickness of the both of coatings was about 6~7 μm (Fig. 1d and f), and no statistic difference in the thickness could be observed. As the mean diameter of the Mg(OH)_2_ particles was 750~850 nm (Supplementary Fig. S1), Mg(OH)_2_ particles confirmed by SEM-EDS with size of less than 1 μm inside the composite coating (in black circles, Fig. 1d) indicated that the particles distributed in the coating with few apparent aggregates.

The contact angle tests showed that the freshly prepared HF sample was super-hydrophilic with a small contact angle of less than 5°. The PLLA coating exhibited hydrophobicity with a contact angle of 78.7 ± 2.1°, while the addition of Mg(OH)_2_ particles reduced surface hydrophobicity, as the contact angle of HM/PLLA dropped to 65.2 ± 3.6°.

### *In vitro* degradation tests

**Figure 2:**
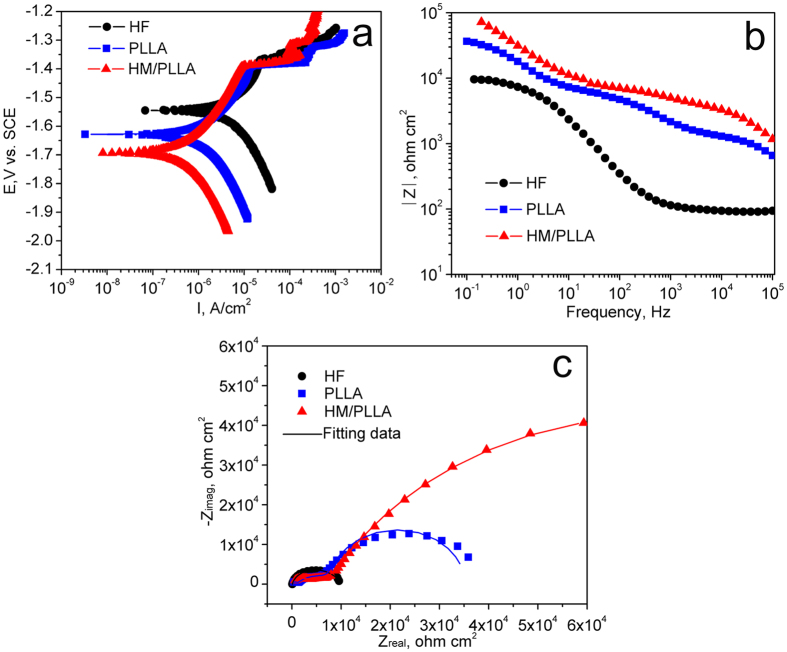
Electrochemical results of JDBM with different coatings. (**a**) Polarization curves; (**b**) Bode plots; (**c**) Nyquist plots.

Electrochemical results of JDBM samples with various coatings are shown in Fig. 2. The natural corrosion current (I_corr_) data calculated using Tafel fitting from the polarization curves (Fig. 2a) of HF, PLLA and HM/PLLA samples are 4.16 ± 0.48 μA/cm^2^, 1.25 ± 0.08 μA/cm^2^ and 0.52 ± 0.12 μA/cm^2^, respectively. The PLLA coatings slowed down the degradation of the magnesium substrate, as I_corr_ values dropped by ~70%, compared to the results of HF samples. The HM/PLLA coating further lowered the natural corrosion current by ~58%.

**Figure 3:**
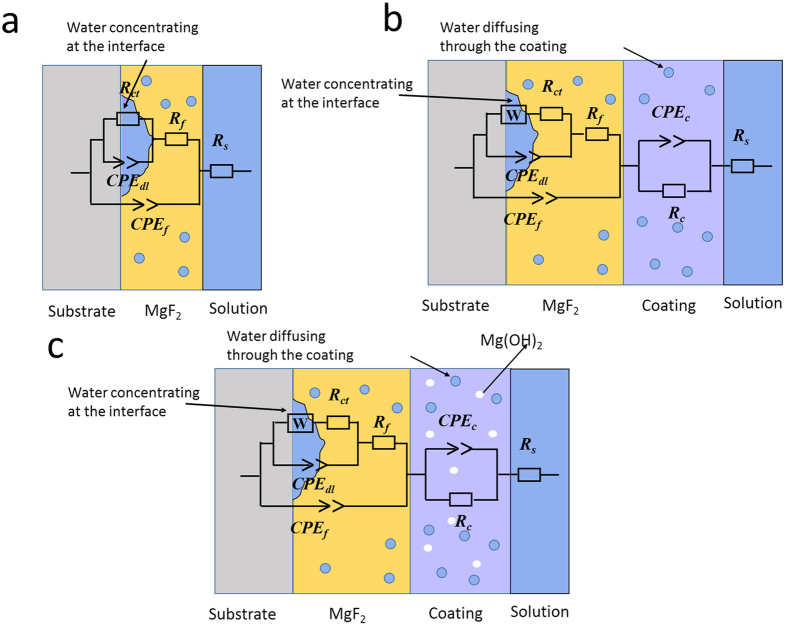
Equivalent circuits (ECs) for fitting EIS spectra. (**a**) EC for HF samples; (**b**) EC for PLLA and (**c**) EC for HM/PLLA samples.

The EIS spectra (Fig. 2b and c) illustrated that PLLA and HF/PLLA specimens presented different impedance responses from those of the HF-treated specimens. The method of electronic circuit models in elucidating the corrosion mechanism of coated specimens in details has been used by many researchers^9^,^26^,^27^,^28^, and in the present work, equivalent circuits were utilized to fit the EIS results. It should be noted that the equivalent circuits are theoretical models resulting from mathematic deduction without considering the practical dispersion effect. However, dispersion effect cannot be ignored in experimental spectra. Therefore, constant phase element *CPE* components (with impedance defined as Z = 1/[Y (I*ω)^n^] were used instead of pure capacitances in the equivalent circuits^29^. The Nyquist plots of the HF samples exhibited two constants while the samples with PLLA or composite coatings displayed three. As a result, different equivalent circuits (Fig. 3) were applied to fit the experimental spectra of different coating samples to obtain more detailed analyses and information of the protective mechanism of the coatings.

Figure 3(a–c) corresponds to the equivalent circuit of HF, PLLA and HM/PLLA samples. Regarding the HM/PLLA samples, as the particles distributed inside the coating to be an integral coating, the electrochemical properties of the composite coating could be characterized using the same electrical element as PLLA coating. Here *R*_*s*_ was the solution resistance; *R*_***f***_ and *CPE*_***f***_ were applied to describe the properties of the polymer coatings; *CPE*_*b*_ and *R*_*b*_ characterized the capacitance performance and the resistance property of the barrier MgF_2_ layer, respectively. Due to the existence of defects in the polymer and the MgF_2_ layer, the solution can infiltrate through the coatings, and once the solution reached and interacted with the substrate, the charge transfer process occurred^30^ and an electric double-layer formed. The characterizations of the double layer were described by a *CPE*_*dl*_ and a *R*_*ct*_ in parallel with it. In addition, in the initial stage of immersion, the infiltration process of the electrolyte into the substrate through the polymer coating and the MgF_2_ layer successively was a slow diffusion reaction due to the relatively compact structure of the coatings. This diffusion characteristics could be described using a W element.

The fitting results are listed in Table 1. The total resistance of HF samples (R_b_ + R_ct_) mainly came from the barrier MgF_2_ layer, while the protective effect of polymer-coated groups was mostly attributed to the resistance of the polymer coatings, and their total resistances (R_f_ + R_b_ + R_ct_) were significantly greater than those of HF samples. It was noteworthy that the addition of Mg(OH)_2_ imposed a great influence on the electrochemical properties of PLLA coating-the resistance improved from 3.38E4 Ω cm^2^ to 8.23E4 Ω cm^2^. Furthermore, the R_ct_ value also increased from 1249 Ω cm^2^ to 3592 Ω cm^2^, indicating a retarded charge transfer process, most probably due to the existence of Mg(OH)_2_ particles.

**Figure 4:**
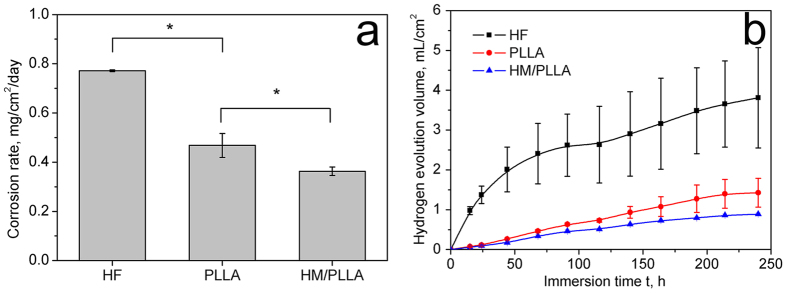
Corrosion test results of JDBM with different coatings. (**a**) Corrosion rate calculated from weight loss test; (**b**) hydrogen evolution profiles; *p < 0.05.

The results of corrosion rate measured from weight loss during static immersion process is displayed in Fig. 4a, and hydrogen evolution results in Fig. 4b. It can be seen from Fig. 4a that the PLLA coating reduced the corrosion rate of HF samples significantly, from about 0.77 ± 0.01 mg/cm^2^/day to about 0.47 ± 0.05 mg/cm^2^/day. The addition of Mg(OH)_2_ further decreased the weight loss rate to 0.36 ± 0.02 mg/cm^2^/day. These results were in accordance with those obtained from hydrogen evolution test (Fig. 4b). During the 10 days of immersion, the volume of evolved H_2_ from polymer-coated specimens, especially the composite coating group, was remarkably less than that of HF samples, further demonstrating the improved corrosion resistance by the composite PLLA/Mg(OH)_2_ particles coating.

**Figure 5:**
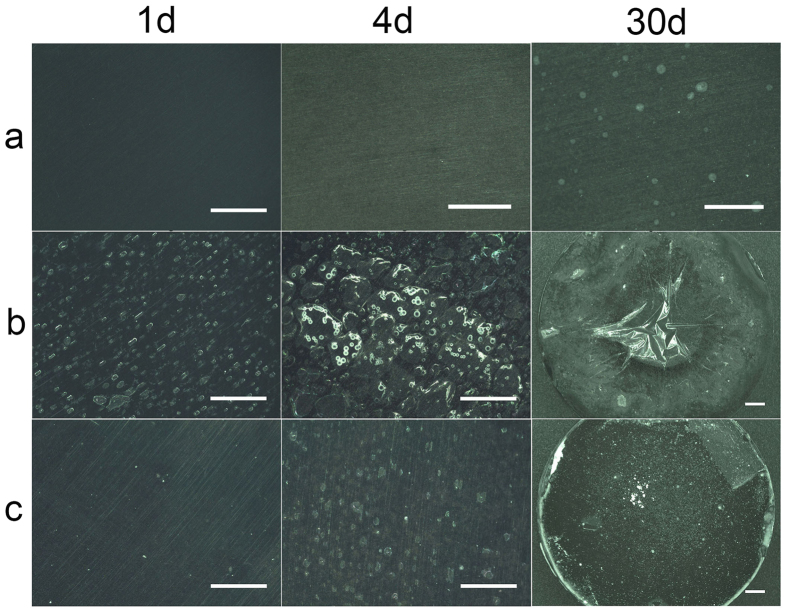
Surface morphologies of different coatings after immersion in DMEM with 10% FBS for different time. (**a**) HF samples, the surface kept flat during the entire immersion process, unless some white spots observed at day 30; (**b**) PLLA, with immersion time increasing, gas pockets formed and grew, leading to delamination of the coating; (**c**) HM/PLLA, within 30 days of immersion, the composite coating kept flat without gas pockets; scale bar represents 1 mm.

Figure 5 shows the optical images revealing surface morphologies of HF, PLLA and HM/PLLA-coated samples after immersion for 1 day, 4 days and 30 days in cell culture medium (DMEM with 10% FBS), respectively. The surface of the HF samples kept flat during the entire immersion process, unless some white spots observed at day 30. For PLLA-coated specimen, after 1 day of immersion, there were a multitude of small bubbles distributing uniformly on the surface, and after immersion for 4 days, quite a few of the tiny bubbles grew larger and merged to form gas pockets, thus delaminating the coating from the matrix. As a result, at the end of the immersion (30 days), most part of the coating peeled off the magnesium alloy substrate, while in the case of HM/PLLA, the condition was significantly alleviated. At day 1, bubbles were barely observed and the coating appeared intact. After 4 days of immersion, only a few small bubbles began to emerge, with both smaller size and lower quantity than those of PLLA-coated specimens. At day 30, the entire coating still adhered onto the substrate tightly, with only a small region at the edge detached from the substrate.

### Direct cell adhesion and proliferation

**Figure 6:**
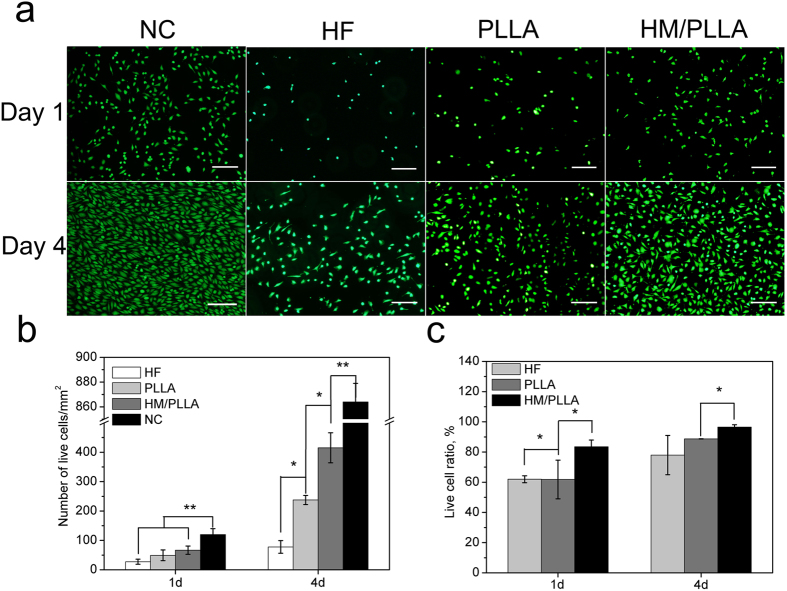
The results of cell proliferation of ECs. (**a**) Live ECs on NC group, HF, PLLA and HM/PLLA samples after culture for 1 day and 4 days, scale bar represents 200 μm; (**b**) statistical analysis of live ECs density and (**c**) live cell ratios on various samples (*p < 0.05, **p < 0.01).

Direct response of human umbilical vein endothelium cells (ECs) to PLLA and HM/PLLA coatings was evaluated by culturing ECs on various samples for 1 day and 4 days (Fig. 6a), with live cell density quantitatively and statistically analyzed (Fig. 6b). While the highest cell density of the NC group were observed, only a few live ECs adhered onto HF samples, and on samples coated with PLLA and HM/PLLA coatings, the adhesive cell density increased distinctly with no statistic difference between the two groups. When the culture period was extended to 4 days, ECs proliferation was recorded on all the samples, and the proliferation rate differed. Cells of the NC group exhibited the highest proliferation rate, from 120 ± 19 cells/mm^2^ at day 1 to 864 ± 15 cells/mm^2^. On HF samples, the cell density increased from 28 ± 9 cells/mm^2^ to 78 ± 22 cells/mm^2^, while the value increased significantly from 49 ± 18 cells/mm^2^ to 238 ± 15 cells/mm^2^ and from 67 ± 14 cells/mm^2^ to 415 ± 51 cells/mm^2^ on substrates with PLLA and HM/PLLA coatings, respectively. It is noteworthy that the proliferation rate on HM/PLLA samples was only slightly lower than that of the NC group, displaying greatly improved cell adhesion and proliferation in comparison with PLLA coating. Furthermore, the live cell ratio (Fig. 6c) on HM/PLLA coating were higher than that on PLLA coating at both day 1 and day 4. Specifically, at day 1, the live cell ratios of PLLA and HM/PLLA were (61.8 ± 12.8)% and (83.5 ± 4.4)%, and at day 4, the ratios were (88.7 ± 0.1)% and (96.5 ± 1.56)%, respectively. These results confirmed the HM/PLLA coating represented significantly more desirable interfacial conditions for the viability and proliferation of ECs, demonstrating superior cytocompatibility with the addition of Mg(OH)_2_ particles into PLLA coating.

## Discussion

Hydrofluoric acid treatment is a frequently used method to improve the corrosion resistance and biocompatibility of magnesium-based implants by *in*-*situ* forming MgF_2_ conversion layers, however, it fails to produce long-term stable coatings in ionic solutions^21^,^31^. Therefore, it is usually applied as a pre-treatment method and further surface modifications like polymer deposition coatings are required for long-term application. As a commonly used polymer coating for protecting magnesium and magnesium alloys, PLLA exhibits improved corrosion resistance and biocompatibility, nevertheless, the interaction between the polymer and Mg alloy substrate, as well as the hydrogen gas generated from the degradation process, often undermined the protection effect of the coating. Thus, new strategies to modify the polymer coating are essential to be developed for clinical implant applications.

In this study, Mg(OH)_2_ submicron particles were added into PLLA to modify polymer coating properties, and the influence of the Mg(OH)_2_ particles on the corrosion protection and the cytocompatibility were systematically investigated. The electrochemical results (Fig. 2) demonstrated the superiority of the HM/PLLA coating in protecting the Mg substrates. The HM/PLLA-coated samples exhibited the lowest natural corrosion current and the highest total resistance (Table 1, R_f_ + R_b_ + R_ct_ for polymer-coated samples, and R_b_ + R_ct_ for HF-treated ones), indicating the greatest resistance against corrosion among the three different surface modifications. More specifically, compared with PLLA coating, the introduction of Mg(OH)_2_ particles increased the resistance of the polymer R_f_ as well as the value of R_ct_, implying the charge transfer process was impeded and the corrosion reaction occurred at the interface of MgF_2_/Mg was slower than that of the PLLA-coated substrate. The results of *in vitro* degradation tests (Fig. 4) confirmed that the addition of Mg(OH)_2_ particles further reduced the corrosion rate of the HF samples as compared with PLLA coating, as both the corrosion rate measured from weight loss (Fig. 4a) and the volume of H_2_ evolution (Fig. 4b) of HM/PLLA samples were lower than those of PLLA and HF samples, respectively.

**Figure 7:**
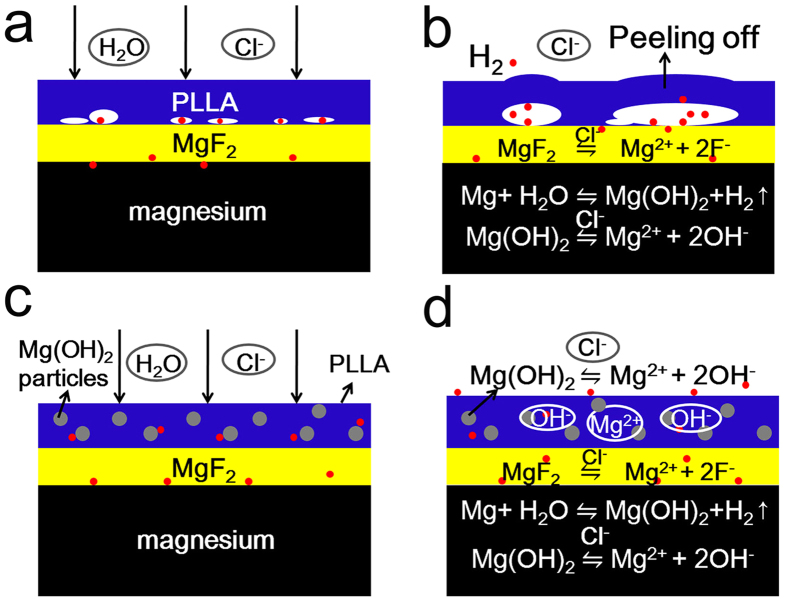
A schematic illustration of the degradation process of polymer-coated samples. The penetrated solution through PLLA coating (**a**) or HM/PLLA coating (**c**) into the interface between the coating and the substrate erodes the Mg substrate and produces H_2_; (**b**) PLLA coating is compact and dense, retarding the release of H_2_ and the H_2_ accumulated at the interface, leading to peeling and delamination of the coating; (**d**) the presence of Mg(OH)_2_ particles deteriorates the density of the polymer coating, providing passages for H_2_ release, thus avoiding the formation of gas pockets and the delamination of the composite coating.

As shown in Fig. 7, a schematic illustration was proposed to depict the influence of the addition of Mg(OH)_2_ particles on the degradation process. For the PLLA samples, the degradation process was supposed to involve the following steps:

In the initial stage of immersion, the electrolyte penetrated or diffused through the PLLA coating and the MgF_2_ layer and reached the interface of MgF_2_/Mg, thus the Mg substrate reacted with water according to the eq. (1).

Due to the existence of Cl^−^, some of Mg(OH)_2_ formed at the interface of MgF_2_/Mg was gradually dissolved, leading to the release of OH^−^ to the vicinity, resulting in local alkaline environment, as shown in (eq. 2).

In addition, though the MgF_2_ layer is generally considered insoluble in water, it can be gradually dissolved in Cl^−^ containing solution^23^ according to the following equation:


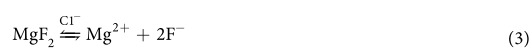


Mg^2+^ released from this reaction disrupted the dissolution equilibrium of eq. (2), so the reaction goes on reversely to form Mg(OH)_2_ in the alkaline vicinity:


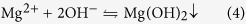


Combining the eqs (3) and (4), that is


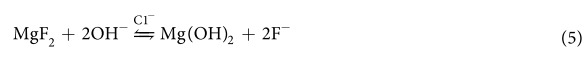


The eq. (5) indicates conversion of the MgF_2_ layer to Mg(OH)_2_.

Due to the barrier layer of the dense and hydrophobic PLLA coating, the diffusion of the water molecules and the ions were retarded, thus the above corrosion reaction rates were considered slower than that without polymer coating. Therefore, the polymer-coated Mg substrate was better protected and degraded slower than HF samples. However, the PLLA coating could also hinder the fast generated H_2_ from immediate diffusing to the surrounding medium, thus once the generating rate of H_2_ was remarkably higher than the diffusion rate, the H_2_ molecules tended to accumulate at the interface of PLLA/MgF_2_, leading to some scattered gas bubbles as shown in Fig. 7a, and with the growing of H_2_ volume, the gas bubbles integrated to form big gas pockets, resulting in the shedding or peeling-off of the coating from the substrate as shown in Fig. 7b. This interpreted the peeling-off phenomenon of PLLA coating in Fig. 5.

The addition of 2% Mg(OH)_2_ particles into PLLA coating (Fig. 7c) decreased the corrosion rate of the alloy as was described above. Compared to the PLLA-coated samples, the main distinction of the degradation of specimens with HM/PLLA coating was that the Mg(OH)_2_ particles participated in the overall process. Mg(OH)_2_ were attacked by penetrated Cl^−^ in the electrolyte and could be gradually dissolved according to eq. (2), releasing OH^−^ and Mg^2+^ to the surrounding environment as shown in Fig. 7d, increasing pH in the vicinity of the substrate. The alkaline microenvironment decelerated the dissolution of the product Mg(OH)_2_ deposited on the substrate and also that converted from MgF_2_ layer, thus the forward reaction of the substrate with water (eq. (1)) was also retarded and the Mg-based substrate degraded at a slower rate.

Further research (Supplementary Table S1) showed that the protective effect with Mg(OH)_2_ particles was content-dependent. Addition of 0.5% Mg(OH)_2_ particles into the PLLA coating accelerated the corrosion (from 0.47 ± 0.05 to 0.57 ± 0.03 mg/cm^2^/day), while with the increase of the amount of the Mg(OH)_2_ particles, the corrosion protection effect of the coating was enhanced gradually. The introduction of Mg(OH)_2_ particles might slightly destroy the compactness of the polymer coating, which is adverse for the protection of the PLLA coating. Nevertheless, with respect to corrosion resistance, this deterioration effect can be compensated by the retard of the forward corrosion reaction of the substrate with water according to eq. (1). Therefore, it is expected that there exists equilibrium between the breach of coating compactness and the hindrance of forward corrosion reaction, thus the amount of particles added to the coating plays a crucial role.

From the images of surface morphologies after immersion in cell culture medium (Fig. 5), it appeared no significant peelings or gas pockets of the HM/PLLA composite coating. Two important factors are considered to be contributed to this phenomenon: the generation rate of H_2_ was relatively slower due to the slower substrate corrosion, and the diffusion rate through the composite coating was faster than that through the PLLA coating, as the compactness of the composite coating was somewhat deteriorated by the addition of the inorganic particles. The addition of Mg(OH)_2_ particles reduced the hydrophobicity and increased the water uptake of the PLLA. The infiltrating water caused swelling of polymer, leading to a number of defects/channels inside the polymer coating due to the expansion of the coating volume during the swelling. Therefore, H_2_ could diffuse through the composite coating more easily than through the compact PLLA coating. Thus, H_2_ did not gather to form gas pockets underneath the coating, preventing the coating from peeling off the substrate. No detachment or peeling-off of the coatings from the substrate is a most fundamental requirement in the application of medical implants.

In addition, the introduction of magnesium hydroxide particles further enhanced the cell adhesion and proliferation as compared to the bare PLLA coating. As the cytotoxicity assay with the extracts of samples with polymer or composite coating (shown in Supplementary Fig. S2) showed similar cell viability of grade 1 (>75%), the higher cell proliferation on HM/PLLA coating most probably resulted from more favourable surface properties, in our study, e.g. the flatter surface during degradation, where fewer gas bubbles formed due to the relatively lower corrosion rate as well as higher H_2_ diffusion rate. On the contrary, with the occurrence of many bubbles under the PLLA coating, some part of the coating delaminated from the substrate, thus it became difficult for the cells to attach and grow on the surface. Furthermore, in the case of the composite coating, the local pH and concentration of Mg^2+^ would be more close to the physiological condition due to slower Mg degradation with the protection of HM/PLLA coating, which is also likely to benefit cell adhesion and proliferation.

As reported in previous works^32^,^33^,^34^, in the blood vessel healing process, the neighbouring ECs migration or the circulating endothelial cells (CEC) recruiting process are assumed to be beneficial for the fast endothelialisation of the vascular. The composite coating modified the magnesium alloy to be a physio-chemical condition which attracted more ECs and enhanced their proliferation, thus accelerating the regrowth of the endothelial layers. In a word, the addition of Mg(OH)_2_ particles into the PLLA coating did not only protect the substrate from rapid corrosion, but also provide a much more desirable interfacial environment for adhesion and proliferation of ECs. This study provides a new strategy to further enhance the corrosion resistance and biocompatibility of magnesium alloy for medical applications.

## Conclusion

A composite PLLA coating with Mg(OH)_2_ particles was prepared on Mg alloy substrate, with the Mg(OH)_2_ particles uniformly distributing in the polymer coating. The *in vitro* degradation tests showed the protective effect of PLLA was improved with the introduction of Mg(OH)_2_ particles, as the degradation rate significantly decreased. A model was proposed to understand the degradation process and mechanisms of corrosion protection of the HM/PLLA composite coating. The analyses indicated that the improvement of the protection most probably resulted from Mg(OH)_2_ particles in the coating retarding the dissolution of the corrosion product Mg(OH)_2_ on the Mg alloy substrate and impeding the forward corrosion reaction of Mg with water, i.e. the degradation of the matrix. Additionally, the introduction of Mg(OH)_2_ particles reduced the damaging effect of the bubbling phenomena occurred with bare polymer coating, improving the adhesion between the coating and the magnesium alloy substrate. Furthermore, the HM/PLLA coating, where fewer gas bubbles formed due to slower corrosion rate and higher H_2_ diffusion rate, presents a more favorable surface for ECs adhesion and proliferation, showing excellent cytocompatibility. The addition of Mg(OH)_2_ particles into PLLA coating on Mg-based substrate appears to be a promising strategy to modify the biodegaradable magnesium-based medical devices for enhanced corrosion resistance and improved biocompatibility.

## Materials and Methods

### Sample preparation

The composition and the processing of Mg-Nd-Zn-Zr alloy (JDBM) alloy could be found in ref. 23. Φ12 × 3 mm, Φ15 × 3 mm and Φ19 × 3 mm disks were used in this study for different measurements. All the specimens were polished with SiC paper progressively up to 3000 grits, ultrasonically cleaned in acetone and ethanol for 10 min, respectively, and dried with warm air. Thereafter, the specimens were pre-treated by 40 wt.% hydrofluoric acid for 24 hours at room temperature to form a compact and protective MgF_2_ layer of ~1.5 μm thickness^23^ on the surface.

PLLA (-(CHCH_3_COO)_n_-) with an weight-average molecular weight of ~100, 000 g/mol was used as the coating material. A PLLA solution was prepared by dissolving the polymer in dichloromethane (analytical grade) at a concentration of 2 wt.%. Prior to use, magnesium hydroxide particles (purchased from Aladdin, China) with an average particle diameter of 750~850 nm (Supplement Fig. S1) were ultrasonically dispersed in DCM for at least 1 hours to obtain uniformly dispersed solution. Then a small amount of the dispersed solution was added to the PLLA solution to obtain a dispersion solution with a Mg(OH)_2_/PLLA ratio of ~2 wt.%. The PLLA and Mg(OH)_2_/PLLA coatings with magnesium hydroxide particles were prepared by a dip-coating method, simply dipping the samples into the solutions for 120 s followed by pulling them out at a constant rate to eliminate the residual solvent at room temperature and the dipping process was repeated 3 times. Then the samples were transferred to a vacuum oven for further drying at 30 °C for at least 48 h.

### Characterization of the polymer coatings

The morphologies and the thickness of the polymer coatings in this study were observed by a scanning electron microscopy (SEM, Hitachi S4800, Japan) equipped with energy dispersive X-ray spectroscopy (EDS). The cross-sections of the coated samples were prepared by imbedding the samples in PMMA and polishing with SiC paper progressively up to 7000 grits. Prior to SEM observation, the samples were coated with a thin layer of gold for better conductivity with a sputter coater (Hitachi E-1045, Japan).

Static contact angle (CA) test was performed to evaluate the surface wettability of the HF, PLLA and HM/PLLA samples using the sessile drop method with an optical contact angle system (OCA20, Germany). A minimum of 6 values of contact angle were collected per group of samples.

### *In vitro* degradation tests

Electrochemical corrosion measurements, static immersion tests and hydrogen evolution experiments were performed in conventional simulated body fluid (c-SBF)^35^ at 37 °C, pH 7.40 for evaluating the influence of the introduction of Mg(OH)_2_ particles on the protective effect of PLLA coating for magnesium matrix.

Electrochemical impedance spectroscopy (EIS) and potentiodynamic polarization curves were measured in the c-SBF with an advanced electrochemical system (Princeton PARSTAT 2273, USA), equipped with a three-electrode cell featuring a Pt counter electrode and a saturated calomel reference electrode (SCE). A surface area of 0.5 cm^2^ for each sample was exposed to the solution during the tests. After immersion in the SBF at open circuit potential (OCP) for 1 h to reach a steady state, EIS tests were conducted at OCP from 100 mHz up to 10^5 ^Hz, with an amplitude of ±10 mV. The impedance date fitting was performed using a Zsimpwin software. Potentiodynamic polarization experiments were carried out from −0.3 V vs. OCP at a scan rate of 1 mV/s, and the measurement ended once the polarization current reached 1 × 10^−3 ^A.

Immersion tests were carried out in accordance with ASTM G31-72^36^ (the ratio of surface area to solution volume was 1 cm^2^:30 mL). The initial weight of the samples was recorded prior to immersion. After immersion in c-SBF for 10 days, the samples were cleaned with boiling chromate acid (200 g/L CrO_3_ + 10 g/L AgNO_3_) for 1 min to remove the surface corrosion products followed by drying with warm air. The dried samples were weighed and the corrosion rates were calculated according to ASTM G1-03.

The hydrogen evolution test was carried out as depicted in ref. 37. The samples were suspended in beakers containing c-SBF with a funnel placed over the specimens to collect hydrogen from the specimen surface. A burette was mounted over the funnel and filled with solution. In this way, the volume of the evolved hydrogen was measured.

In order to better simulate the *in vivo* environment containing proteins and some other organic molecules, Φ19 × 3 mm disks were immersed in Dulbecco’s modified eagle medium (DMEM, Gibco, USA) containing 10% fetal bovine serum (FBS, Gibco, USA) in a cell incubator and the ratio of medium volume to sample surface area was 1 mL/1.25 cm^3^, according to ISO 10993-12. The medium was replaced periodically. After immersion for 1, 4 and 30 days, the coating morphologies were observed with a stereo microscope.

### Direct cell adhesion assay

The human umbilical vein endothelium cell line EA.hy926, was purchased from the Cell Bank, Chinese Academy of Sciences. The cells were cultured in DMEM supplemented with 10% FBS in a cell incubator (37 °C, 5% CO_2_ and 95% humidity). Cells were subcultured every 2–3 days. Disk samples were placed in a 24-well plate and 1 mL of 3 × 10^4^/mL cell suspension was added to each well. Cells seeded in a blank well served as negative control (NC). After 1 d and 4 d of incubation, Live/Dead staining (LIVE/DEAD® Viability kit, Thermo Fisher Scientific, US) was conducted according to the manufacturer’s protocol, and the stained cells were observed by an inverted fluorescence microscopy (IX 71, Olympus). The adhesion assays were carried out in triplicate and repeated at least twice.

### Statistical analysis

The results were expressed as the means ± standard deviations. Difference between two groups were tested using the Student’s test. For variance between different groups, one-way ANOVA was performed and statistical significance was defined as p < 0.05.

## Additional Information

**How to cite this article:** Shi, Y.-j. *et al*. Enhanced corrosion resistance and cytocompatibility of biodegradable Mg alloys by introduction of Mg(OH)_2_ particles into poly (L-lactic acid) coating. *Sci. Rep.* **7**, 41796; doi: 10.1038/srep41796 (2017).

**Publisher's note:** Springer Nature remains neutral with regard to jurisdictional claims in published maps and institutional affiliations.

## Supplementary Material

Supplementary Materials

## Figures and Tables

**Table 1:** The fitting results of EIS spectra of different coating samples.

	Rs (Ω cm^2^)	Y_f_ (Ω^−1^ cm^−2^ s^n^)	n	R_f_ (Ω cm^2^)	Y_b_ (Ω^−1^ cm^−2^ s^n^)	n	R_b_ (Ω cm^2^)	Y_dl_ (Ω^−1^ cm^−2^ s^n^)	n	R_ct_ (Ω cm^2^)
HF	94.1	—	—	—	1.02E-5	0.88	8245	1.51E-4	1	1522
PLLA	100.1	6.67E-9	0.82	3.38E4	1.45E-6	0.71	5015	1.37E-5	0.91	1249
HM/PLLA	103.3	1.86E-8	0.72	8.23E4	6.94E-7	0.73	4055	7.89E-6	0.80	3592
