# Supplementary material for: Enhanced corrosion resistance and cytocompatibility of biodegradable Mg alloys by introduction of Mg(OH)2 particles into poly (L-lactic acid) coating
Source: Sci Rep. 2017 Feb 2;7:41796. doi: 10.1038/srep41796 (PMC5288779; doi:10.1038/srep41796)
Supplement: Supplementary Materials [file srep41796-s1.doc]

**Enhanced corrosion resistance and cytocompatibility of biodegradable Mg alloys by introduction of Mg(OH)_2_ particles into poly (L-lactic acid) coating**

Yong-juan Shi^1,*^, Jia Pei^1,*^, Jian Zhang^1^, Jia-lin Niu^1^, Hua Zhang^1^, Sheng-rong Guo^2^, Zhong-hua Li^3^，Guang-yin Yuan^1,**^

^1^ National Engineering Research Centre of Light Alloy Net Forming and State Key Laboratory of Metal Matrix Composite, Shanghai Jiao Tong University, Shanghai 200240, China.

^2^School of Pharmacy, Shanghai Jiao Tong University, Shanghai 200240, China.

^3^Microport Endovascular(Shanghai) Co., Ltd, Shanghai, 201318, China

*These authors contributed equally to this work.

**The corresponding author: Prof. Guangyin Yuan, School of Materials Science and Engineering, Shanghai Jiao Tong University, Shanghai, 200240, China

[Tel: +86-21-34203051](tel:+86-21-34203051); Fax: +86-21-34202794

E-mail: [gyyuan@sjtu.edu.cn](mailto:gyyuan@sjtu.edu.cn)

**Methods**

**Measurements of the size of the Mg(OH)_2_ particles.**

In order to obtain uniform distribution of Mg(OH)_2_, Mg(OH)_2_ particles were firstly dispersed physically in DCM ultrasonically for at least 1 h after stirred for 30 min. The particle size in the dispersion was measured using a Particle Analyzer, and the result was shown in Supplementary Figure S1. Additionally, one drop of the dispersion was dropped onto a silicon wafer, and after evaporation of the solvent, the particles were observed using a SEM, as shown in the inserted image in Figure S1.

**Cell viability assay.**

The Φ19×3 specimens were sterilized by ultraviolet light for 30 min and the extracts were prepared according to ISO10993-12 using DMEM containing 10 % FBS .The surface area of extract medium ratio of 1.25 cm^2^ mL^-1^ and the extract time was 72 h. Then the extracts were sterile filtered using a 0.2 μm syringe filter.

The cytotoxicity of the extracts were detected by Cell Counting Kit-8 (CCK-8). EA.hy926 were seeded on 96-well plates in a density of 2500 cells/100 μL per well. The cell culture served as negative controls and those without cells as blank controls. After 24 h pre-cultivation under cell culture conditions for 24 h to obtain optimal cell adhesion, the cell culture medium was replaced with the prepared extracts. The cells were further incubated for 2, 4, 7 days, respectively, 10 μL of CCK-8 solution was added to each well and the 96-well plate was incubated at the incubator for 1 h. After that, the OD value was measured on a microplate reader at a wavelength of 450 nm to determine the cell viability. Six wells were prepared for each group of extract in one assay and the assay was repeated three times. The cell viability was calculated as following:

Cell viability (%) =(OD(experiment)-OD(blank))/(OD(control)-OD(blank))×100

**Influence of Mg(OH)_2_ content on the protection of the composite coating.**

PLLA coatings with different Mg(OH)_2_ contents were prepared on HF-JDBM substrates as described in the section of Sample preparation in the manuscript. Immersion test was performed, and the calculated weight loss rate was displayed in Supplementary Table S1.

**Water uptake test**

PLLA and HM-PLLA films were prepared by using an automatic coating machine, and the solvent was evaporated by air drying overnight, followed by vacuum drying up until constant weight was obtained. The final film thickness was about 150 μm and then these films were cut into 10×10 mm^2^ samples. Water uptake experiments were performed at 37℃ in pH 7.40 c-SBF. At 3 d and 10 d, the specimens were taken out from the solution, cleaned by ultrapure water, wiped at the surface and weighed. Then the specimens were vacuum dried at room temperature until constant weight was obtained. The water uptake was calculated according to

Water uptake ratio (%)=
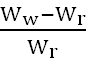
×100%

where W_w_ represents the weight of the wet sample after wiping and W_r_ represents the weight of the sample after drying.

**Supplementary Figures**

**Supplementary Figure S1.** The size distribution of Mg(OH)_2_ particles in DCM after [ultrasonic dispersion](http://www.baidu.com/link?url=HxVm1dYSz0X-HrySRSeZ7eAsBDAZHCHdujkT1eZQ7uxN8ds8dByuu4wfExtTKRrHI13JeZHGlCeCv8126DVNDDZURkqRmWfbLfZ8QwAHLclc1eKVvt2DtFDBvlauctrr) and electromagnetic stirring successively. The inserted is a SEM image of a single Mg(OH)_2_ particle. The diameter of Mg(OH)_2_ particle is confirmed to be about 750~850 nm. The results showed that the particles distributed uniformly without aggregation.


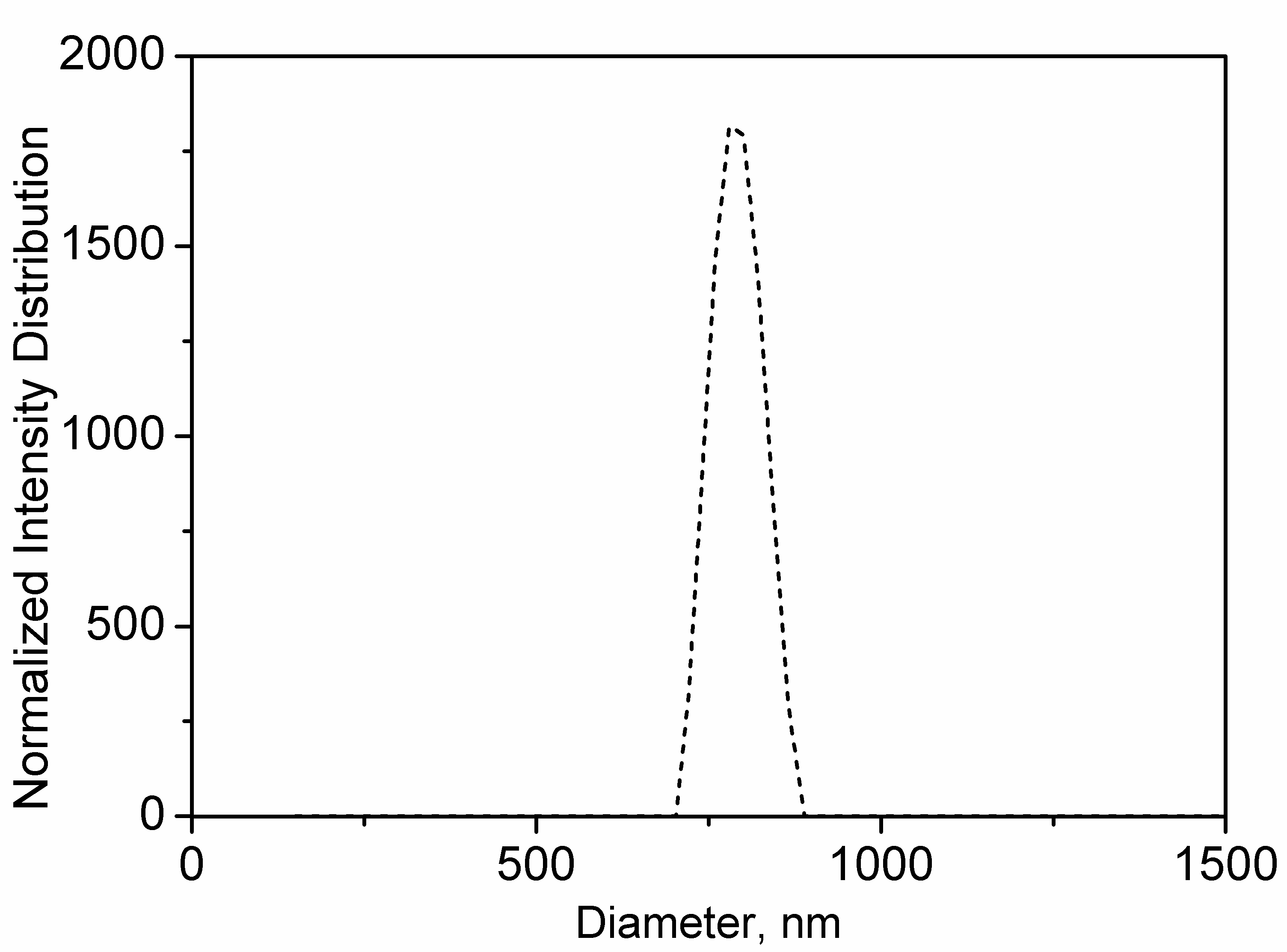

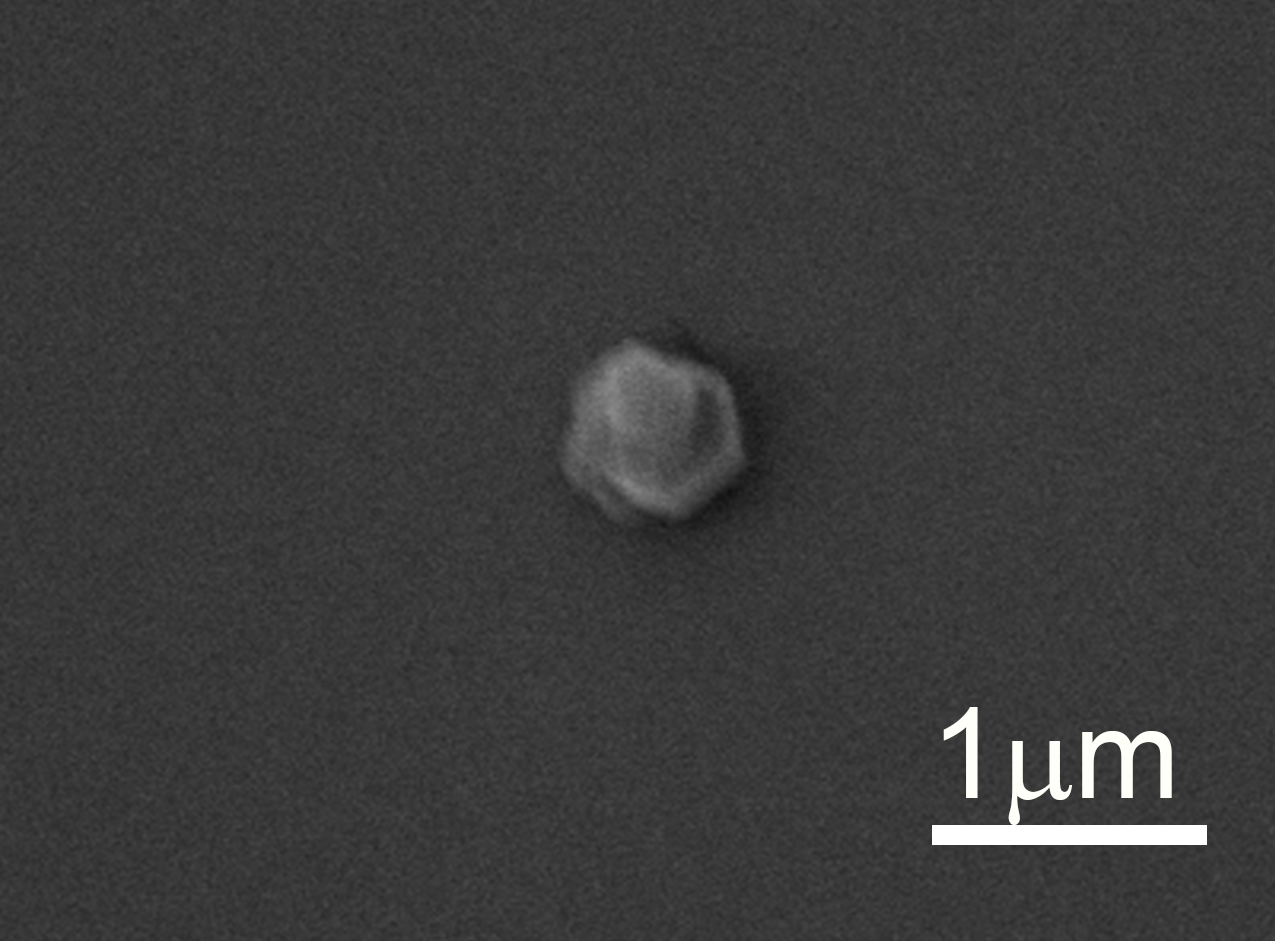


**Supplementary Figure S2.** EA.hy926 viability after 2, 4 and 7 days' culture in extraction media of HF-JDBM with PLLA and HM/PLLA coating, respectively, using a CCK-8 assay. The cell viability for the three groups of extracts during the 7 days of culture ranged from 85% to 100% compared to the control. As a result, the cytotoxicities of the HF-JDBM and polymer coated samples are Grades 0-1(>75%), indicating that the coatings meet the in vitro cytocompatibility requirement for implants application. In addition, the supplement of Mg(OH)_2_ particles showed no deterioration of cell viability.


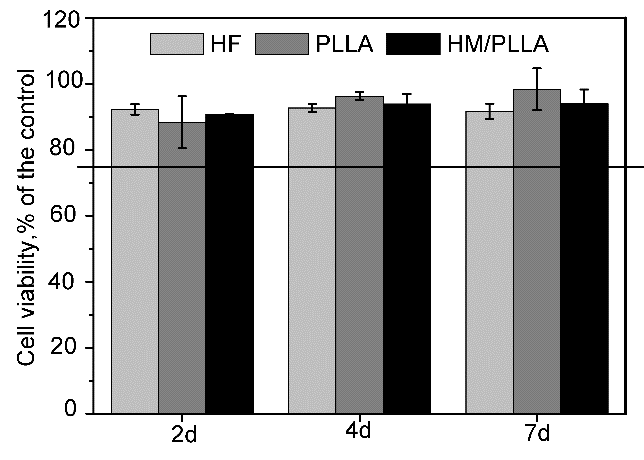


**Supplementary Figure S3.** Water uptake of PLLA and HM-PLLA after degradation for various time. The water uptake ratio of the HM-PLLA almost doubled that of the PLLA film during the immersion, and the water uptake ratio of both two films increased with immersion time.


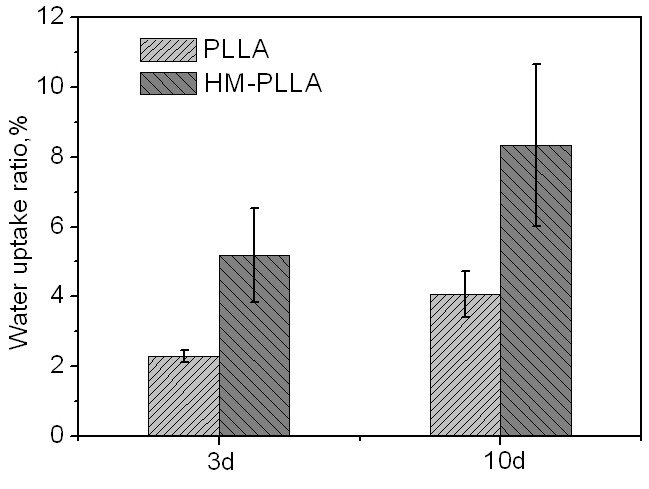


**Supplementary Tables**

**Supplementary Table S1** The effect of contents of Mg(OH)_2_ particles on the corrosion rate of the substrates.

| Mg(OH)_2_ content | 0% | 0.5% | 1% | 2% |
| --- | --- | --- | --- | --- |
| **Corrosion rate (mg/cm^2^/day)** | 0.47 ± 0.05 | 0.57 ± 0.03 | 0.39 ± 0.01 | 0.36 ± 0.01 |

[1] Y. Han, Z. Fan, Z. Lu, Y. Zhang, S. Li, In vitro Degradation of Poly [(L‐lactide)‐co‐(trimethylene carbonate)] Copolymers and a Composite with Poly [(L‐lactide)‐co‐glycolide] Fibers as Cardiovascular Stent Material, Macromolecular Materials and Engineering, 297 (2012) 128-135.
